# Measuring Psychological Strains: A Psychometric Study to Develop a Shortened Version of the Psychological Strain Scales

**DOI:** 10.3390/bs14121157

**Published:** 2024-12-02

**Authors:** Ching Sin Siau, Jie Zhang, Won Sun Chen, Nur Zakiah Mohd Saat, Bob Lew, Xiaodong Ma, Kairi Kõlves

**Affiliations:** 1 Centre for Community Health Studies, Faculty of Health Sciences, University Kebangsaan Malaysia, Kuala Lumpur 50300, Malaysia; chingsin.siau@ukm.edu.my (C.S.S.); nurza@ukm.edu.my (N.Z.M.S.); 2 School of Public Health, Shandong University, Jinan 250012, China; 3 Department of Sociology, State University of New York Buffalo State, Buffalo, NY 14222, USA; 4 Faculty of Health Sciences, Curtin University, Bentley, Perth, WA 6102, Australia; sharon.chen@curtin.edu.au; 5 Australian Institute for Suicide Research and Prevention, School of Applied Psychology, Griffith University, Brisbane, QLD 4122, Australia; boblew@asiacrux.com (B.L.); k.kolves@griffith.edu.au (K.K.); 6 Faculty of Medicine, Pingdingshan University, Pingdingshan 467000, China; maxiaodong@pdsu.edu.cn

**Keywords:** psychological strains, psychometric properties, validity, China

## Abstract

The Psychological Strain Scale (PSS) has been widely used in various populations to assess the risks of suicidality and mental disorders. The current study aims to shorten the original 40-item scale and test its psychometric properties. Data were derived from three samples in China: (1) undergraduate students (*n* = 10,742), (2) non-medical office employees (*n* = 1044), and (3) hospital workers (*n* = 949). A questionnaire was administered to the samples at about the same time of the year 2017. Data were randomly divided into Sub-study 1 (70% of the sample) and Sub-study 2 (30% of the sample). In Sub-study 1, principal component analyses were run and five items with the highest factor loadings within each subscale were retained, resulting in a 20-item PSS scale. Cronbach’s α estimates were above 0.70. In Sub-study 2, confirmatory factor analyses for the three samples revealed RMSEA values below 0.10, PNFI values below 0.50 for all samples, and CFI, TLI and NFI values above 0.90 for the student and non-medical office employee samples, but the hospital worker sample had a TLI of 0.88. The PSS-20 and its subscales were significantly associated with past-year suicidal ideation. The PSS-20 has acceptable psychometric properties but requires further testing in university students and hospital workers.

## 1. Introduction

Suicide and suicidality are important public health issues. Of note, while suicide rates have declined dramatically during the past 20 years, 17% of suicide deaths globally in 2019 occurred in China [1]. University students suffer from high suicidal ideation prevalence, which was reported at 18% in one study [2]. About 6% of hospital staff reported recent suicidal ideation, while a study reported 4.2% of Chinese workers reported prior 12-month suicidal ideation [3,4].

Understanding the etiologies of suicidality and assessing at-risk individuals in various populations could aid in preventing suicide, as they allow for root-cause and early interventions. One of the relatively new theories which attempted to understand the etiology of suicidality and psychopathologies is the Strain Theory of Suicide (STS). This theory was first conceptualized by Zhang and Song and Zhang et al. [2,5,6,7], and was built on earlier sociological frameworks regarding criminal behavior [8,9]. While strain theory in relation to crime suggests that social and psychological strains cause violence directed outward [9], the strain theory of suicide posits that these same types of strains lead to violence directed inward when the accumulated psychological tension is released.

The STS proposes that individuals experience conflicting life events, known as psychological strains. Strains are different from stress in the sense that strains consist of at least two conflicting stressors within an individual, causing psychological tension, frustration and suffering [10]. There are four types of strains (or sources), which are the Value, Aspiration, Deprivation, and Coping strains. The first type of strain is the Value strain, in which an individual experiences strain in the area of values, such as the conflict between traditional and modern values. The second type of strain is the Aspiration strain, which refers to an individual’s experience of discrepancy in their aspiration vs. reality. An example is when an individual wishes to be successful, and yet experiences many obstacles in life. In terms of the Deprivation strain, an individual experiences it when they perceive a relative deprivation in comparison with others who originate from the same background. For example, perceiving that most people around one are living in a better and more comfortable environment may be a source of deprivation strain. The last type of strain is the Coping strain, which refers to an individual’s perceived deficiency in coping with life’s challenges, such as feeling out of control and not able to catch up when dealing with everyday life [10]. The greater the strains experienced by an individual, the higher the likelihood of an individual to experience psychopathologies and suicidality [7,11]. The STS was employed in various contexts, including in a recent discourse analysis on public tweets on suicide [12].

In order to examine the psychological strains that affect mental health, a multidimensional questionnaire, the Psychological Strain Scales (PSS), was constructed [10,13]. The development of the PSS began in 2012 with an initial pool of around 160 items generated by 30 researchers from China and the US. Forward- and backward-translation ensured that the Chinese and English versions of the items were similar. The questionnaire was refined through validity and reliability analyses through data obtained in two separate studies with college students [10]. Zhang and Lyu [14] also validated the English version of the scales among US university students.

The PSS has been particularly used to measure psychological strains as conceptualized by the Strains Theory of Suicide, and to our best knowledge, there are no other scales measuring this concept, although the term psychological strain has been liberally used to describe psychological distress, anxiety or burnout in past studies [15,16]. The PSS has been employed in a number of studies. For example, recent findings show that religious affiliation in Chinese students could contribute to greater strain and suicidality [17]. During the COVID-19 pandemic, psychological strains were found to positively indirectly influence suicidal ideation via brooding and depression in Pakistani university students [18]. Psychological strains were also tested in other populations, such as among athletes [19].

However, given the current length of the full-scale questionnaire, the PSS-40 requires a longer time to administer. In addition, a longer scale may incur participant fatigue. Therefore, it is necessary to shorten the scale in order to ensure the practicality of its application and interest in its usage across various settings. Since strains are often measured in relation to other psychological outcomes, a shorter scale will enable the PSS to be administered along with a battery of other scales [20]. However, shortening the scale may have some disadvantages. For example, removing some of the items may lead to reducing the coverage of a particular concept, or losing the original multifactorial domains of a scale [20]. Huen et al. developed the short form of the PSS (PSS-SF) using the Item Response Theory approach [7]. Five items from the original 40 items were retained, which were Items 9, 12, 14, 16, and 20. The PSS-SF demonstrated concurrent validity with psychache and suicidality, in addition to achieving adequate internal consistency reliability and confirmatory factor analysis fit indices. However, due to the short-form nature of the scale, the domains of the Value, Aspiration, Deprivation, and Coping strains were not retained [7]. The retention of these domains is important as the scale could possibly differentiate the types of strains experienced by individuals from different backgrounds, and thus more accurate interventions could be proposed to deal with these different types of strains. Therefore, the aim of this study is to shorten the PSS-40, a 40-item scale to 20 items, retaining the four factors and testing its psychometric properties. This includes testing (1) the factor structure for the PSS-20 in the four subscales of the Value, Aspiration, Deprivation, and Coping strains, (2) the concurrent validity of the PSS-20 with suicidal ideation in the prior 12 months, and (3) the internal consistency reliability of the scale and subscales.

## 2. Materials and Methods

### 2.1. Study Design

This is a cross-sectional study to establish the validity and reliability of the shortened Psychological Strain Scales-20. A cross-sectional design was selected as this design is cost-effective and feasible [21]. In addition, the cross-sectional design is able to fulfil the objectives of this study.

### 2.2. Participants and Data Collection

Participants were either college students or workers aged between 18 and 60 years old. For college students, they had to be under enrolment in a university in China. For workers, they had to be employed. Individuals who were either unemployed or not enrolled in a college, or were unable or unwilling to provide informed consent, were excluded.

The surveys were carried out in various settings and areas in China. The first involved non-medical office employees in urban areas in Beijing, China, and selected medical workers from Qilu Hospital in Shandong, China [11]. The second survey was among college students from seven provinces in China. We selected one university from each of the following locations: Jilin, Qinghai, Shandong, and Shaanxi provinces; Ningxia and Xinjiang autonomous regions; and the municipality of Shanghai [22]. A questionnaire including the PSS was administered to the samples at about the same time of the year 2017. After obtaining informed consent, the questionnaires were distributed to the participants. No more than 20 min were required to complete the questionnaire.

### 2.3. Measures

The variables tested in this study are psychological strain (with its sub-domains of the Value, Aspiration, Deprivation, and Coping strains) and prior twelve-month suicidal ideation. The following measures were used to measure these variables.

#### 2.3.1. Psychological Strain

The Psychological Strain Scale (PSS) measures psychological strains experienced by the participants based on four sources of strain: Value strain, Aspiration strain, Deprivation strain, and Coping strain [14]. Each subscale consists of 10 items, leading to a total of 40 items in the scale. The participants answer on a five-point Likert scale ranging from 1 = “never, it’s not me”, 2 = “rarely, it’s not me”, 3 = “maybe, I’m not sure”, 4 = “often, it’s like me” to 5 = “yes, it’s exactly me”. Higher scores of each subscale indicated greater strain in a particular area of strain, with scores ranging between 40 and 200. The total score of all subscales constitutes the total strain experienced. The Cronbach’s alpha coefficients for the total PSS-40 in this study were 0.956 in university students, 0.969 in hospital workers, and 0.968 in non-medical office employees.

#### 2.3.2. Suicidal Ideation

Suicidal ideation in the prior 12 months was used to test the concurrent validity of the PSS-20 and its subscales. Among college students, 12-month suicidal ideation was obtained from the Suicidal Behaviors Questionnaire-Revised (SBQ-R) [23]. Only one item was used from the SBQ-R. The participants answered Item 2, “How often have you thought about killing yourself in the past year?”, and those who responded “never” were considered as not having suicidal ideation in the past 12 months, while those who responded “rarely—1 time”, “sometimes—2 times”, “often—3 to 4 times”, and “very often—5 or more times” were considered as having suicidal ideation in the past 12 months. We coded not having suicidal ideation as “0” and having suicidal ideation as “1”.

Twelve-month suicidal ideation among workers was obtained from the life-event history section of the National Comorbidity Survey (NCS) interview [24]. The participants answered “Yes” = 1 or “No” = 0 to the occurrence of prior 12-month suicidal ideation.

### 2.4. Data Analysis

Statistical analysis was conducted using IBM SPSS Statistics for Windows, version 27.0 (IBM Corp., Armonk, NY, USA) and IBM SPSS Amos, version 24.0 (IBM Corp., Meadville, PA, USA). Statistical significance was set at *p* < 0.05.

A pooled dataset consisting of *N* = 12,735 participants was created by combining the three samples of university students, hospital workers, and non-medical office employees. Using the random selection function in the SPSS, the pooled dataset was randomly split into two datasets in order to conduct exploratory factor analysis (EFA) and confirmatory factor analysis (CFA) on separate datasets. Dataset one (Sub-study 1) contained 70% of the participants (*n* = 8920), whilst dataset two (Sub-study 2) contained the remaining 30% of the participants (*n* = 3815).

Descriptive analysis (frequency, percentage, mean/median, and standard deviation/inter-quartile range) was conducted to examine the demographic variables and scale properties. Data normality was determined using skew and kurtosis values of ±2 and ±7, respectively [25].

#### 2.4.1. Factor Structure

In Sub-study 1, the validity of the scale was examined. EFA involving principal component analysis (PCA) using varimax rotation was employed. PCA is often used to reduce the number of dimensions to primary (or principal) components while maintaining the majority of the original information [26]. The Kaiser–Meyer–Olkin measure of sampling adequacy should be above 0.60, while the Bartlett’s test of sphericity should be significant [27]. In the first EFA conducted on the PSS-40, the extraction was constrained to four factors, corresponding to the four factors of the original PSS-40. The top five items that loaded into each factor were extracted, while the remaining five items were discarded, resulting in a total of 20 items that made up the tentative PSS-20. Further analysis on the 20 items was conducted through a second EFA to examine the factor structure of the PSS-20. The total variance explained by the factors should be more than 60% [28]. Items with a factor loading value of >0.40 are deemed acceptable [29], and item-to-total correlation should be >0.50 [30]. Pearson’s correlation was used to analyze the relationship between the sub-domains of the PSS-20. Inter-factor correlation should ideally be between >0.30 and <0.70 [31].

In Sub-study 2, via a confirmatory factor analysis, model fit indices were evaluated. The model fit indices employed in a confirmatory factor analysis of this study included chi-square-value/degree of freedom (χ^2^/*df* < 5.00) [32,33], normed fit index (NFI ≥ 0.95) [34], comparative fit index (CFI ≥ 0.95) [28], Tucker–Lewis index (TLI ≥ 0.95) [35], parsimonious normed fit index (PNFI ≥ 0.50) [36], root mean square error of approximation (RMSEA < 0.01 (excellent), < 0.05 (good), and < 0.08 (mediocre) [37]. Confirmatory factor analyses were conducted for the remaining 30% of the samples (i.e., the overall, student, hospital worker, and non-medical office employee samples).

#### 2.4.2. Concurrent Validity

Concurrent validity was analyzed using logistic regression to identify associations between the PSS-20 and subscales with suicidal ideation in the prior 12 months. The results are reported in odds ratios (*OR*s). Data from Sub-study 1 were used.

#### 2.4.3. Internal Reliability

To examine the internal consistency reliability of the newly formed PSS-20 scale, Cronbach’s alpha coefficient was employed. The examination of internal consistency reliability estimates was conducted on Sub-study 1. Items contributing to a Cronbach’s alpha of more than 0.70 were retained, whereas items contributing to a low coefficient were excluded from further analysis [38,39].

#### 2.4.4. Ethical Considerations

This study was conducted in accordance with the Declaration of Helsinki, and approved by the Institutional Review Board of Shandong University (Approval No.: 20161103). The information sheet included counseling service hotlines available in each province, allowing students to reach out for support if they experienced any psychological distress during or after completing the questionnaire.

## 3. Results

There was a total of 12,735 participants in the pooled dataset of university students, hospital workers, and non-medical office employees. The mean age was 22.9 (SD = 6.23, range = 18 to 60 years old). There were 7909 (62.1%) females and 4826 (37.9%) males. A majority were university students (*n* = 10,742, 84.4%), followed by non-medical office employees (*n* = 1044, 8.2%) and hospital workers (*n* = 949, 75%) (see Table 1).

### 3.1. Sub-Study 1

#### 3.1.1. Validity Analysis

EFA using PCA was conducted on the randomly selected 70% of the pooled sample. Factorization, using varimax rotation, revealed an acceptable Kaiser–Meyer–Olkin measure of sampling adequacy (KMO = 0.97), which is above the recommended value of 0.60. Bartlett’s test of sphericity was significant (χ^2^(780) = 198,275, *p* < 0.001). The diagonals of the anti-image correlation matrix of all items were above 0.50 (range of 0.90–0.98), thus supporting the inclusion of each item in the factor analysis. All items had communalities ≥0.40 (range of 0.40–0.63; see Table 2), suggesting reasonable factorability. Given these overall indicators, factor analysis was performed on all 40 items of the PSS-40. A cumulative variance of 53.7% was obtained. The top 5 items which had the largest factor loadings in each factor were chosen. This included items 4, 5, 6, 7, and 9 from the Deprivation factor; 5, 6, 8, 9, and 10 from the Coping factor; 4, 7, 8, 9, and 10 from the Aspiration factor; and 4, 5, 6, 7, and 10 from the Value factor. These items were further examined as agreed by the content experts.

A second EFA examined the 20 items selected. The cumulative variance had increased to 64.1%, which was higher than the recommended minimum of 60%. All 20 items loaded into their respective factors (i.e., Value, Aspiration, Deprivation, and Coping). All items had primary loadings above 0.40 (range of 0.59–0.81; see Table 2). The Aspiration factor had the largest explained variance, at 42.3%, whilst the Value factor had the smallest (6.4%). Factor loadings in the Aspiration factor ranged between 0.775 and 0.614, and were 0.769–0.717 for Coping factor, 0.805–0.588 for the Deprivation factor, and 0.764–0.606 for the Value factor. The correlation between all factors was reflected in Table 3, and all relationships were significant at *p* < 0.001, with *r* coefficients ranging from 0.470 to 0.615 for the overall Sub-study 1, from 0.450 to 0.612 for the university student sample, from 0.567 to 0.706 for the hospital worker sample, and from 0.556 to 0.715 for the non-medical office worker sample. Communalities were between 0.532 and 0.728 (see Table 2).

#### 3.1.2. Concurrent Validity

The results of the logistic regression conducted on the Sub-study 1 sample showed that the scales and subscales of the university students’, hospital workers’ and non-medical office employees’ samples were significantly associated with suicidal ideation in the prior 12 months. Scale scores of the PSS-20 and its sub-domains predicted higher odds of suicidal ideation in the *OR* range of 1.047–1.197. In university students and hospital workers, the highest odds for suicidal ideation were associated with coping strain (*OR*: 1.186 and 1.197, respectively), whilst in non-medical office workers, the Deprivation strain predicted the highest odds (*OR*: 1.168) (see Table 3).

#### 3.1.3. Reliability Analysis

For Sub-study 1, internal consistency reliability estimates for the scale and subscale scores for the overall sample, and the university student, hospital worker and office employee sample were above α = 0.70 (see Table 4). Item-to-total correlations ranged between 0.497 and 0.784 (see Table 3).

### 3.2. Sub-Study 2

#### Confirmatory Factor Analysis

**Figure 1:**
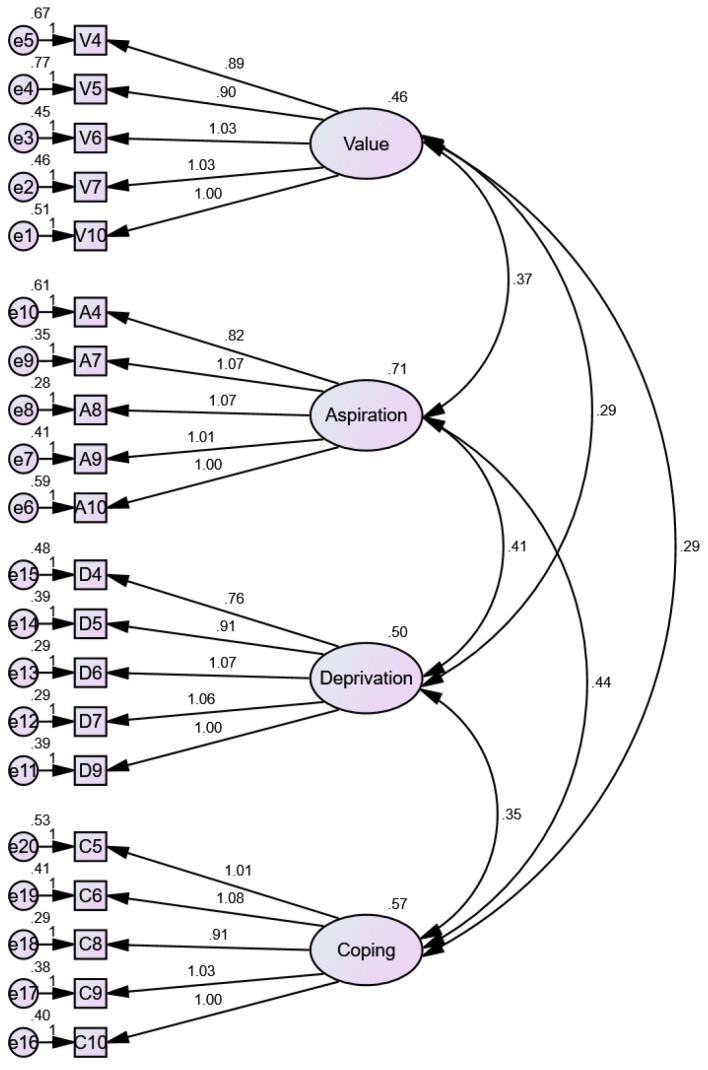
Confirmatory factor analysis on the Sub-study 2 data (*n* = 3815).

Using the 20 selected items from the PSS-40, we performed CFA to test the model fit for the overall, university students’, hospital workers’, and non-medical office employees’ samples, which is the remaining 30% of the pooled sample. The results showed that in terms of χ^2^/*df* value, only the hospital worker and non-medical office employee samples met the <0.05 cut-off. The RMSEA values were all below 0.10, and the PNFI values were all above 0.50. The CFI, TLI and NFI values were above 0.90 for the student and non-medical office employee samples, but the hospital worker sample had a TLI of 0.88 (see Table 4, Figure 1).

## 4. Discussion

The Psychological Strain Scale (PSS) measures four strains of the Strain Theory of Suicide [2]. However, the 40-item scale (or the PSS-40) is rather long and time-consuming. Therefore, the aim of this study was to shorten the PSS into 20 items (or the PSS-20) and to test the psychometric properties of the shortened scale whilst retaining the four original domains from the PSS. The samples used consisted of university students, hospital workers, and non-medical office employees from China.

The results of the factor analysis showed that the 20 items selected for inclusion in the new PSS-20 scale loaded adequately to their original domains in the PSS-40, thus retaining the four-factor domains of Value, Aspiration, Deprivation, and Coping. The total variance of 64.1% was higher than the recommended 60% in social science studies [28]. Therefore, the structural validity of the PSS-20 as a four-domain questionnaire was adequate.

We had mixed findings for the confirmatory factor analysis results. The requirements for absolute fit indices, such as χ^2^/*df* were not met for the overall and student populations, but were met for the hospital worker and non-medical office employee samples. χ^2^/*df* may not be a suitable model fit index for large samples, such as the Sub-study 2 overall (*n* = 3815) and university student sample (*n* = 3231), as larger sample sizes tend to find statistically significant values between model observations and observed data for statistics that are χ^2^-based. The RMSEA values are less susceptible to the effect of large sample sizes; therefore, it might be a more accurate indicator of fit for our large sample [40]. The RMSEA values of 0.066, 0.067, and 0.070 for overall, student, and non-medical office employee samples were adequate, while for hospital workers (0.097), these were marginally short of the cut-off of <1.00, above which the fit is considered poor.

Relative fit indices, such as the NFI, TLI, and CFI, showed that all values for the overall, student, and non-medical office employee samples were between 0.93 and 0.94, falling slightly short of the cut-off of 0.95, suggesting reasonable fit. However, the relative fit indices for the hospital worker population were consistently below the accepted cut-offs. Finally, in terms of the PNFI, the values were above the recommended cut-off, suggesting the four-factor model to be parsimonious. This may be due to the nature of the Strain Theory of Suicide, which limits the number of strains into four psychosocial strains.

Pearson’s correlation conducted between the subscales revealed that the *r*-coefficients were below 0.70 for the overall and sub-samples (range of 0.454 to 0.666). This suggests that the domains were not unidimensional to form a general construct, and were sufficiently differentiated from each other. However, the correlation coefficients between aspiration and deprivation among non-medical office employees and hospital workers were strong.

In measuring the concurrent validity between PSS-20 and its subscales with suicidal ideation, logistic regressions obtained significant *OR*s of more than 1.00, suggesting that the higher the strain experienced, the higher the odds of the participant experiencing suicidal ideation in the last 12 months. This was true for the overall and all the sub-samples of this study. The findings indicate that psychological strains measured by the PSS-20 are associated with suicidal ideation, conforming to the theory proposed by Zhang et al. that higher levels of strains could lead to higher levels of suicidality [6]. The positive association between psychological strains and suicidal ideation was found elsewhere, for instance, in a study on Pakistani university students [18]. The association between suicidal ideation and psychological strains may make the PSS-20 useful for assessing suicidality. However, it needs to be noted that a different item was used to measure 12-month suicidal ideation for the student and worker samples. This could lower the robustness of the conclusions we draw.

In terms of the internal consistency reliability measured using Cronbach’s alpha, the PSS-20 and its subscales achieved α > 0.70 across the overall and all sub-samples. Therefore, this scale can be reliably employed for the student, hospital worker and non-medical office employee samples. With regard to the item-to-total correlation, all items correlated above >0.50 with the total of their respective subscales, indicating that all items are measuring the same constructs within their domains. However, an item (“I don’t know if women should have the same rights that men do”) had a relatively low item-to-total correlation of 0.497. Another study by Zhang et al., which validated the PSS-40, similarly found that this was a weak item (*r* = 0.386). This may be due to conflicts in gender roles not being a distinguishing feature in the value strains felt by the Chinese population [41].

Overall, the psychometric properties of the PSS-20 were found to be adequate for usage among non-medical office employees. However, CFA model fit indices for the student and hospital worker samples appear to be lacking, pointing to potential issues in structural validity in the PSS-20 for these populations, such as limitations in capturing the latent strain dimensions among university students and hospital workers. While items in the PSS are sufficiently general to cater to a wide range of populations (e.g., the item “I wish I could achieve the highest goal in my life, but I cannot” could apply to hospital workers and university students alike in describing their Aspiration strain), there may be considerations that are specific to certain population contexts. For example, in terms of Aspiration strains, university students in China may be concerned about getting a job in the context of high unemployment rates [42]. Hospital workers, on the other hand, may be concerned about the diversification of their job scope to include involvement in academic research and publication for promotion purposes [43]. Therefore, the authors of the PSS may consider whether different versions of the scale are necessary for various populations.

Ongoing validations of a scale, especially one that is conceptually tied to a particular societal value, are common with the passage of time [44]. Future studies examining the strain construct may involve qualitative interviews of individuals from university student and hospital worker populations on the particular strains faced by them, in accordance with their contexts. Hence, the psychometric rigor of the PSS-20 or alternate forms of the scale may benefit from further testing among the student and hospital worker populations.

There are a few strengths and limitations to this study. First, the large sample size enabled adequate power to conduct the estimation procedures. Secondly, we used samples from a few backgrounds, such as university students, hospital workers, and non-medical office employees. The limitations are that the sample size for the students was much larger than that of the hospital workers and non-medical office employees. This will limit the generalizability of the results to non-student populations, especially hospital workers who are sampled from only one hospital. We only sampled our participants from urban areas. In addition, we used a different scale to estimate suicidal ideation in the prior 12 months between the student sample and the hospital worker/non-medical office employee samples. Another limitation was that the data were collected in 2017, and therefore future studies should compare our study results with analyses based on more recent data. Future studies could also explore other strain models for the university student and hospital worker population.

This study has practical implications in reducing the length of the PSS-40 to 20 items, thereby decreasing participant fatigue, non-response rate and missing data in questionnaire surveys involving the measurement of psychological strains. In addition, being more user-friendly, the scales could be used in larger scale or in follow-up studies in a time- and cost-effective manner. Even though the scale has limited validity among student and hospital worker populations, it could now be administered to office workers in studies concerning organizational health, assessing strains in workers from various industries and their associations with mental health. Following the results of this study, future exploration of psychological strains among university student and hospital worker populations may reveal unique strains faced by these populations, thereby enriching the current knowledge of this topic.

In conclusion, the current study aimed to shorten the PSS-40 scale while retaining its four domains: Value, Aspiration, Deprivation, and Coping strains. Tested on a diverse population comprising university students, hospital workers, and non-medical office employees, the results of the EFA using principal component analysis identified five items from each domain, forming the new PSS-20. A subsequent EFA revealed a four-factor solution consistent with the original PSS-40. The CFA results demonstrated adequate model-fit indices in the non-medical office worker sample but were suboptimal in the university student and hospital worker samples. Cronbach’s α ranged from adequate to excellent for the scale and its subscales, while concurrent validity was established through the scales’ positive association with suicidal ideation. The shortened PSS-20 enables more time- and cost-efficient studies on psychological strains, although further testing is recommended in university student and hospital worker populations.

## Figures and Tables

**Table 1:** Demographic characteristics of study participants (*N* = 12,735).

Variable	Sub-Study 1 (*n* = 8920)	Sub-Study 2 (*n* = 3815)	Total (*N* = 12,735)
	*n* (%)	*n* (%)	*n* (%)
Sex			
Male	3342 (37.5).	1484 (38.9)	4826 (37.9)
Female	5578 (62.5)	2331 (61.1)	7909 (62.1)
Occupation			
University student	7511 (84.2)	3231 (84.7)	10,742 (84.4)
Hospital worker	675 (7.6)	274 (7.2)	949 (7.5)
Non-medical office employee	734 (8.2)	310 (8.1)	1044 (8.2)
Age (mean ± SD)	22.94 (6.26)	22.88 (6.15)	22.92 (6.23)

**Table 2:** Explained variance, factor loadings, and communalities based on a principal component analysis with varimax rotation for the PSS-20 in Sub-study 1 (*n* = 8920).

Item No.	Item	Explained Variance (%)	Factor Loading	Communality	Item-to-Total Correlation
	Total	64.082			
	Factor 1: Aspiration	42.307			
9	I wish I could be successful, but there are too many obstacles in my life.		0.775	0.722	0.757
8	I wish I could achieve the highest goal in my life, but I cannot.		0.764	0.736	0.784
7	I wish I could change my current living condition, but I cannot.		0.762	0.710	0.756
10	I wish I had fewer burdens in my life, but I have to deal with so many responsibilities every day.		0.738	0.670	0.706
4	I wish I had more power in my life, but I cannot realize it according to some reasons.		0.614	0.532	0.610
	Factor 2: Coping	8.079			
6	Even with small problems, I sometimes feel low and cannot get going.		0.769	0.696	0.718
9	When I have a problem, I always stay alone and away from others.		0.749	0.679	0.695
8	When I have difficulties in what I am doing, I usually give up the task.		0.726	0.681	0.691
5	I cannot forget unpleasant experiences, and the more I think, the worse my feelings are.		0.724	0.636	0.661
10	In dealing with things, I often feel out of control and not able to catch up.		0.717	0.646	0.685
	Factor 3: Deprivation	7.271			
5	I cannot go to church as much as people around me can, because I am poor.		0.805	0.728	0.689
4	My family does not have the money to support me to go to school.		0.789	0.681	0.605
6	I have the same qualities as some of my colleagues, but they are paid much more than I am.		0.662	0.664	0.712
7	Most people around me have better and more comfortable working environment.		0.648	0.661	0.706
9	Compared to others, it is more difficult for me to make money.		0.588	0.575	0.638
	Factor 4: Value	6.426			
6	Between traditional and modern values, I don’t know what I should follow.		0.764	0.648	0.636
7	Between chastity and sexual liberty, I don’t know what I should do.		0.712	0.596	0.602
5	I don’t know if women should have the same rights that men do.		0.690	0.534	0.497
4	My parents and my best friends (peers) sometimes have different views on certain things, and I always find it difficult to deal with them.		0.619	0.474	0.518
10	The traditional values are always opposite to what I have learned from school, I cannot make a choice what to believe.		0.606	0.548	0.582

**Table 3:** Characteristics of the PSS-20 scale, subscales, and 12-month suicidal ideation for the overall, university student, hospital worker, and non-medical office employee samples in Sub-study 1 (*n* = 8920).

Variable	Mean	Standard Deviation	Skew-Ness	Kurtosis	Cronbach’s α	Correlations (Pearson’s *r*)					Suicidal Ideation (*OR*)
						1	2	3	4	5	
Overall Sub-study 1 sample											
PSS-20 (1)	46.33	12.85	0.16	0.18	0.926	1	0.767 ***	0.864 ***	0.810 ***	0.813 ***	1.054 ***
Value (2)	11.34	3.68	0.46	0.27	0.787		1	0.549 ***	0.512 ***	0.470 ***	1.106 ***
Aspiration (3)	12.65	4.47	0.23	−0.39	0.885			1	0.601 ***	0.615 ***	1.151 ***
Deprivation (4)	10.75	3.65	0.53	0.48	0.856				1	0.554 ***	1.115 ***
Coping (5)	11.59	3.94	0.41	0.17	0.866					1	1.188 ***
University student											
PSS-20 (1)	46.23	12.59	0.15	0.27	0.922	1	0.758 ***	0.859 ***	0.799 ***	0.810 ***	1.054 ***
Value (2)	11.29	3.63	0.48	0.38	0.780		1	0.535 ***	0.500 ***	0.450 ***	1.102 ***
Aspiration (3)	12.61	4.40	0.23	−0.34	0.878			1	0.577 ***	0.612 ***	1.151 ***
Deprivation (4)	10.64	3.58	0.54	0.58	0.855				1	0.539 ***	1.103 ***
Coping (5)	11.68	3.94	0.39	0.17	0.859					1	1.186 ***
Hospital worker											
PSS-20 (1)	47.65	13.75	0.17	−0.05	0.946	1	0.803 ***	0.884 ***	0.865 ***	0.840 ***	1.056 ***
Value (2)	11.75	3.88	0.40	0.07	0.837		1	0.606 ***	0.567 ***	0.567 ***	1.165 **
Aspiration (3)	12.95	4.59	0.16	−0.57	0.919			1	0.706 ***	0.643 ***	1.117 *
Deprivation (4)	11.57	3.95	0.43	0.16	0.875				1	0.664 ***	1.148 **
Coping (5)	11.38	3.77	0.35	0.14	0.907					1	1.197 ***
Non-medical Office employee											
PSS-20 (1)	46.08	14.45	0.20	−0.41	0.942	1	0.806 ***	0.890 ***	0.858 ***	0.848 ***	1.047 ***
Value (2)	11.44	4.04	0.31	−0.40	0.812		1	0.612 ***	0.556 ***	0.595 ***	1.096 ***
Aspiration (3)	12.78	4.99	0.28	−0.72	0.908			1	0.715 ***	0.650 ***	1.134 ***
Deprivation (4)	11.07	3.93	0.43	−0.08	0.845				1	0.665 ***	1.167 ***
Coping (5)	10.79	3.99	0.68	0.41	0.897					1	1.148 ***

Note. *** *p* < 0.001. ** *p* < 0.01. * *p* < 0.05. *OR* = Odds Ratio.

**Table 4:** Goodness-of-fit indicators for the PSS-20 based on population in Sub-study 2 (*n* = 3815).

Model	Χ^2^ (*df*)	Χ^2^/*df*	NFI	TLI	CFI	PNFI	RMSEA (90% *CI*)
Overall	2883.34 (164)	17.58	0.93	0.92	0.93	0.80	0.066 (0.064, 0.068)
Student	2518.01 (164)	15.35	0.93	0.92	0.93	0.80	0.067 (0.064, 0.069)
Hospital Worker	582.85 (164)	3.55	0.87	0.88	0.90	0.75	0.097 (0.088, 0.105)
Non-medical Office Employee	410.43 (164)	2.50	0.90	0.93	0.94	0.78	0.070 (0.061, 0.078)

Note. *df* = degree of freedom. NFI = Normed Fit Index. TLI = Tucker–Lewis Index. PNFI = Parsimonious Normed Fit Index. RMSEA = root mean square error of approximation. *CI* = confidence intervals.

## Data Availability

Data are available from the corresponding author upon reasonable request.
